# Mr. Saumarez, on the Functions of the Pancreas, &c.

**Published:** 1806-10

**Authors:** Richard Saumarez

**Affiliations:** Kennington, Surry


### Medical and Phyfical Journal.

VOL. XVI.] OCTOBER, 1806. [NO. 92.

*Printed for R. PHILLIPS, by W. Thorne, Red Lion Court, Fleet Street, London.*

### *To the Editors of the Medical and Phyfical Journal.*

GENTLEMEN,

IN looking yesterday over the Medical Journal of the present month, I noticed a physiological Inquiry, by Dr. Rush, of Philadelphia, into the functions of the spleen, liver, and pancreas.

1. To the spleen he supposes the office belongs of becoming a receptacle or reservoir to the blood, whenever it is suddenly or preternaturally excited from the stimulus in excess either of exercise, intemperance, or passions of the mind; that without this provision, which the Almighty in the formation of man has so beneficently made, a state of plethora would constantly ensue.

2dly, The liver he supposes receives the blood from every part of the system, in order to subject that part of it, which had not been completely animalized, or divested of its chylous properties, to a secretory process, and afterwards to pour the product of this secretion, mixed with the liquor of the pancreas, into the duodenum, to be absorbed or otherwise taken up by the lacteals, and conveyed with the chyle from the stomach into the blood vessels, in order to be completely converted into red blood.

3dly, The pancreas he conceives resembles the salivary glands, that it pours out its liquor directly upon the hepatic bile in the common duct, before it enters the duodenum, to act upon it in a concentrated state, and thereby to change it into perfect chyle.

With a view to avoid beginning the discussion of these new doctrines in the middle, as Dr. Rush has done, it will be necessary to invert the order in which he has placed them; I shall therefore take a cursory review of the process of digestion, in order that we may, by a natural and legitimate deduction, arrive at the use for which these organs are designed to subserve.

By the organs of sense the food is selected, by the teeth it is comminuted, by the mouth it is masticated, by the saliva it is blunted and blanded, and, finally, by the active energy of the stomach and the fluid it secretes, this food becomes digested and assimilated to the nature of the system in which it is received. The gastric juice has the power not only of destroying the sensible qualities of common matter, but of animating it; not only of killing living, but of vivifying it anew; it loses the living properties it originally possessed, and becomes assimilated to the specific nature of the system by which it is received.

It is probable that in vegetables, and in the lower order of animals, the quantity of food received, corresponds to the wants and waste of their respective systems, and to the power of the organs by which that aliment is to be assimilated. On the contrary, in the higher order of brutes, and especially of man, when he acts like a brute, such is the voracity of their appetite and the comparative weakness of their organs, that they are constantly led to take not only substances that are unapt and unfit to be digested, but to devour a much larger quantity than is either necessary for the support of the system, or than the limited powers of the digestive organs can assimilate; a necessity, therefore, evidently appears for the existence of auxiliary means, not only with a view of affording to the stomach a larger quantity of blood, for the formation of gastric or assimilating juice, at those times when it is particularly wanted, but as means of separating the digested from the undigested parts of the aliment, the chylous from the fæculent.

If the whole of the food which the stomach receives were perfectly assimilated, and chyle existed in it pure and unmixed, there would be no necessity for the existence of an intestinal canal, or of auxiliary organs, by the energy of whose fluids a separation of the aliments takes place. I am therefore led to inquire into the fabric of the intestinal canal, the part into which the aliment, which the stomach contained, is received ;—of the pancreas and hepatic system, as the agents by means of which this separation is effected, and, finally, of the Spleen, by which these organs are supplied with a more abundant quantity of blood when their actions are preternaturally excited in the process of digestion.

Where the pyloric extremity of the stomach terminates, the the chylous canal begins; * instead of a smooth and polished surface, it has a folded and corrugated one, by transverse ridges, which have received the appellation of valvulæ conniventes. The area of this organ is wonderfully increased by means of this construction. This increase of surface seems evidently designed for two purposes; — for the purpose of prolonging and retarding the passage of the ingesta in this part of their course, and for the benefit of increasing the space from whence the lacteal vessels may arise. These ridges, in the living system, are erect and rigid, not loose and flaccid, as we behold them in the dead subject; so that the aliment, instead of passing over their angles, is involved within their folds.

As the aliment from the stomach enters the duodenum, it appears in general of a pappy consistence, of a greyish colour, is often striated with a white fluid, and is called *chyme*. The heterogeneous nature of this mass renders it unapt aud unfit to afford nourishment to the blood, whose constant waste it is destined to supply; this part of the system therefore, by an organization the most admirable, is supplied with means, by which the dross may be separated from the pure parts, the fæculent from the chylous.

My limits will not allow me to give an anatomical description of these auxiliary organs, or of the particular quality of the fluids which they secrete; suffice it to say, that at a short distance from the pyloric extremity of the stomach, on the surface of the duodenum, the pancreas and liver terminate by means of two excretory ducts; the excretory duct of the pancreas pierces directly the coats of the duodenum, and finally discharges its contents into it. The excretory duct of the liver has a mode somewhat different in its termination; when it reaches the incurvature of the duodenum, instead of perforating the several tunics of the intestine directly as the pancreatic duct, it runs a short distance within the external coat, before it proceeds through the internal one; when therefore the liver secretes bile, and the peristaltic motion of the intestine is suspended, an accumulation into the hepatic trunk, and perhaps a regurgitation into the pori biliari and branches of the vena portæ, would frequently take place

* I do not think the appellation of the intestinal canal, and subsequent division of it, into small and large intestines, either proper or applicable to the use for which it is designed; the small intestines in fact, receive more matter than the large; I should think it more apt to call that portion of the canal, on which the lacteal vessels arise, the chylous canal; viz. the duodenum, jejunum, and ilium. The other portion in which the feculent matter is received, I would give the appellation of feculent or excrementitious canal, comprehending the cæcum, colon, and rectum.

 if if a reservoir did not exist, into which this bile is received; hepatic bile therefore flows through the ductus cysticus into the gall bladder. Its use seems to be, not so much to do good as to prevent mischief; not so much to furnish a regular supply of bile to the chymous canal, as to prevent regurgitation into the liver. It would therefore seem to be a wrong conclusion which Dr. Rush has made, that "the liver is constantly secreting bile," when the mode in which the excretory duct is constructed, shows its entire dependance on the action of the duodenum, and when the chyme it contains renders these fluids absolutely necessary.

The pancreatic duct either enters the duodenum singly, or forms a ductus communis with the hepatic ducts; when it enters the duodenum singly, its course into it is more direct than the course of the biliary ducts; and when it forms a ductus communis, it commonly opens anteriorly to either of them. So far, therefore, from Dr. Rush's opinion being true, that the pancreas pours out its liquor directly upon the bile, it is the liver that pours out its liquor directly upon the liquor of the pancreas.

The nature of this construction renders it probable that the passage of the pancreatic juice and bile into the chylous canal is very different; whilst there is a constant admission of the one, there must be a frequent interruption to the passage of the other.

After the chyme has passed the ductus communis, two very sensible changes in it are beheld; whilst the contents of these ducts are seen floating over it, the chyme assumes a more chylous appearance, and a precipitation of the pure from the feculent parts are seen to take place, which immediately adhere to the surface of the chylous canal, for the lacteal vessels to absorb.

Let it not be supposed that these are fanciful or hypothetical opinions; since I was led to entertain them, they have been proved by the test of experiment, performed by men of the first abilities in the profession.

Experiment. A dog was fed on animal food, and in three hours the abdomen was opened. A part of the duodenum and jejunum, of considerable length, was cut open, so that the contents might be observed. Portions of food, reduced to a pultaceous mass, were seen oozing through the pylorus; the bile was likewise observed to pass slowly out of its duct, which, when carefully attended to, appeared to flow *over the surface of the digested matter*, adhering to the intestines. Upon removing the bile from the surface surface of this digested matter, it did not appear to ha[illegible] mixed with it in any sensible degree.*

Mr. Astley Cooper has informed me, that by openi[illegible] the stomach and duodenum of living animals, a soluti[illegible] of whatever is digestible, is produced in the stomach; a[illegible] that no sooner is this clear solution mixed with the juic[illegible] from the pancreas and liver in the duodenum, than precipitation is produced. The precipitate is the chyl[illegible] which adheres firmly to the coat of the small intestines; he is therefore led to think, that from his experiments [illegible] living animals, it is the office of the pancreatic juice a[illegible] bile to separate the nutritious from the feculent parts [illegible] the food.

It is very difficult, perhaps it is impossible, to discrim[illegible]nate the separate share which organs like these posses[illegible] which are destined to perform a joint and co-operate o[illegible]fice; we may however infer, from the pure and vital natur[illegible] of the arterial blood, of which the pancreatic juice is pro[illegible]duced, and from the more direct termination of the duc[illegible] itself, that it forms a more active and more constant pre[illegible]cipitant than the fluid produced by the liver, whose secre[illegible]tion is not only the offspring of venous blood, but from th[illegible] oblique nature of whose excretory duct, the fluid it secretes would seem to be entirely subservient to the sympathy which it receives from the chymous canal, at the only time when that fluid is wanted. We may conclude, I say, that the pancreatic juice separates the less feculent parts of the chyle from the most pure, and, finally, that the bile separates the most feculent of the whole from the less offensive part, completing what the pancreatic juice had begun.

If this be the true state of the case, how erroneous must be Dr. Rush's opinion, that "the liver is designed to receive the blood from every part of the body, in order to subject that part of it which had not been completely animalized or divested of its chylous properties to a secretory process, and afterwards to have the products of this secretion, mixed with the liquor of the pancreas and duodenum, to be absorbed or otherwise to be taken up by the lacteals, and conveyed with the chyle *from the stomach* into the blood vessels, in order to be converted into red blood."—When the fact is, that none of these juices, in a state of health, are ever directly applied to the surface of these organs.

* Vide Dr. Saunders, on the Liver, page 123.

 The

The regular peristaltic motion of the chymous canal is from the stomach to the cæcum, and it is ever a perversion of action whenever there is an inversion of motion, whenever bile obtains admission into the stomach, it is the effect of disease, causing sickness, head ach, &c; and whenever it is applied directly to the surface of the chymous canal, it produces, under certain circumstances of predisposition, the jungle fever of the East, the yellow fever of the West Indies, or cholera morbus of Europe; and, finally, when bile is actually absorbed into the system from any part on which it is deposited, whether pori biliarii, gall bladder, or intestines, it produces hepatic mischief, which is manifested by the discolouration of the secretions, and the complexion of the skin, from the pallid or sallow cast to the darkest degree of yellow: So far, therefore, from bile being taken up for a beneficent purpose, and of being conveyed "with the chyle from the stomach into the blood, in order to be converted into red blood;" whenever this absorption does take place, instead of affording assistance to the system by being instrumental in the process of chylification, it produces all the mischief of which bile is capable; it acts upon the blood by the sensible qualities it contains, producing irritation and jaundice: these are the effects that are produced when it is conveyed into the system in general, instead of being expelled out of it.

It is when this morbid condition of parts takes place that Dr. Rush supposes the liver is designed for the necessary purpose of performing the same office as the stomach, in repairing the daily waste; for in cases, he says, "of long fasting or sickness, in which the office of the stomach is suspended, the liver performs a vicarious duty: and when its chylopoetic office is impaired or interrupted, the stomach, by performing its functions with double care, prevents the evils of an abstraction of nourishment from the body."

1. It is very true that the liver is present in nearly all animals, because there are few animals, whose digestive powers are so strong as to be enabled to assimilate the whole portion of the food which they receive, without its assistance; since, however, there is no animal without a stomach, but there are some that exist without a liver, Dr. Rush's conclusion is an erroneous one, that "it is upon a footing with the stomach, and designed to perform an office in the system equally necessary with the stomach to the support of life." Life cannot be long supported without without the power of the stomach; but we know that in many diseased conditions of the liver, when it has ceased to perform its functions, and when it is in fact of no use whatever, the actions of life have been long continued.

2. Much less is it true, "that the immense and disproportionate size of the liver in the fœtus, compared with its size in the adult, is in order that nourishment may be carried on exclusively by that viscus." The liver in the fœtal state is, in no instance, concerned in the process of assimilation more than the stomach itself; the fœtus derives the whole of its nourishment from the mother, thro' the medium of the maternal portion of the placenta; the blood which is effused from thence on the fœtal portion, (and which resembles in some respect chyle) is the power by which it is presumed it becomes assimilated to the nature of the fœtal system, from whence it is absorbed and conveyed by the umbilical veins for the evolution of its frame.

It does not appear to me that Dr. R. has any knowledge whatever of the different degrees of evolution which take place in the different organs of the fœtal frame, or of the end for which this variety is caused. It is therefore proper to tell him, that the organs which are more immediately destined to preserve and perfect the system when the fœtal state ends and the adult begins, are especially distinguished by the rapidity of their growth, and the consequent magnitude of their size. I may enumerate as the first in order,

1. The head with the organs of sense, and the nerves that are connected with them.

2. The mouth, trachea, and lungs.

3. The heart and arterial system.

4. The œsophagus and stomach with its auxiliary organs, namely, the pancreas, hepatic system, and spleen.

When we reflect on the relative weakness of the infant stomach, compared with the indigestible quality of the different articles of food which it is liable to receive; the necessity must be apparent of having auxiliary organs by which chylification may be accomplished, of a strength and magnitude that will prevent the deleterious effects that would otherwise constantly ensue, and not for the design "*that nourishment may be carried on exclusively by that viscus, without any aid from the stomach.*"

It is very true that chyle is found in the blood in an uncombined state after a full meal, not because "it is necessary it should undergo a sceond chylopoetic process in the liver,"

liver," but because a larger quantity has been absorbed by the lacteals, than can be at once assimilated to the nature of blood by the lungs; it must therefore make several circuits through the lungs, before it can be perfectly sanguified and fitted for the support and restoration of parts.

Nothing but a total ignorance of these functions, and of the difference of quality in the blood as it proceeds from the lungs, from what it is when it returns to them, could have led him to assert, "that venous blood, from whence hepatic bile is secreted is less disposed to putrefaction than arterial blood taken from any other part of the body." It is Dr. Rush's misfortune to increase the importance of auxiliary organs, and to degrade those that are primarily essential to the preservation and support of life. Let him only compare the florid and fluid blood as it proceeds from the lungs when it has obtained from them an addition of vital air, and is in consequence rendered fit to answer the purposes of restoration and secretion, with the black moribund gore as it returns to them through the right side of the heart: he would not have ventured to assert, "that venous blood is less disposed to putrefaction than arterial blood, taken from any other part of the body."

From an attentive examination of the phenomena of respiration, and the important benefit which the blood in consequence obtains, I have no hesitation in saying, that the lungs perform a correspondent action upon the air that the stomach does upon the food; that the only difference which subsists between them arises from the difference of the substances which each of them has separately to act upon, to assimilate or digest. Whilst the lungs act upon and assimilate air, with a view of meliorating the blood in point of quality; the stomach acts upon grosser materials, in order that the blood may be supplied in point of quantity; these organs however are means devoted to one end, and perform one and the same office, by acting upon things foreign to the system, and assimilating them to its own nature, for its nourishment and support.*

The excretion of mephitic gas from the lungs, in the act of expiration, (a part of venous blood, which corresponds to the fæculent matter from the intestines, if he has not ascertained the fact by experiment) ought to have taught him that arterial blood is more vital "than venous." It is this

* I must refer the reader to the System of Physiology which I have published, where these subjects are treated at large.

this low degree of vitality which venous blood contains, that renders bile, out of which it is formed, to possess less living, and consequently more sensible properties than either the gastric or pancreatic juices; we therefore find hepatic bile either in the pori biliarii or truncus hepaticus, although possessing sensible properties in a greater degree than either of those fluids, is nearly an inodorous, tasteless, fluid, and colourless. It is however a law in the animal economy, that in proportion as effects proceed from their causes, they not only lose the identity of their character, but the very purity and excellence of their nature. In conformity to this law it is, that the farther hepatic bile proceeds from its source, either when subsisting in the gall bladder, or when united with fæculent matter in the intestinal canal, that it verges from a living to a dying state; that it loses its inoxious, and acquires its sensible and stimulating qualities, that it then becomes intensely bitter to the taste, offensive to the olfactory sense, apparently heterogenous in its nature, of a consistence extremely viscid, sometimes concreting, and forming calculi.

When bile has undergone these changes, it ceases to act as bile is intended to do; instead of depurating the chyme, it unites with it; instead of assisting the progress of chylification, it hurries its total expulsion.

It is to these decomposed qualities which putrid bile possesses, that physiologists in general have ascribed its virtues; that my honoured friend, Dr. Saunders, in his excellent Treatise on the Liver, has ascribed anti-septic power to the bitterness it possesses after it has become putrid, and that its virtues consisted in its being a mild purgative.*

Attention however to the construction of the chylous canal, falsifies the assertion; that canal is especially constructed by the number and transvertion of valvulæ conniventes to *retard* the motion of the chyme along the first part of its course; it is therefore most unreasonable to suppose that the real and direct intention of the hepatic system is to hasten its expulsion; if this were the case, no harmony between these parts could subsist; the action of the chymous canal would always tend to prevent the motion which the bile was always destined to promote, so that the ductus communis, instead of opening in the duodenum,

---

* Dr. Rush himself allows that bile undergoes a process of putrefaction in the gall bladder, from the bitterness it acquires by its stagnation in it.

denum, ought to discharge its contents upon the cæcum, colon, or rectum.

Men who reflect on the physiology of the animated system, and can reconcile this warfare of parts, that are dependent on each other, possess very inadequate and erroneous phantasies of the beautiful harmony and subordination that subsists between the parts of which the whole is composed; it is far more reasonable to conclude that the hepatic system is designed to co-operate with the chymous canal, and not to defeat the particular intention for which it is especially formed; pure healthy bile therefore, recently secreted by the liver, is on that account a bland and mild fluid; after long residence in the gall bladder, or after uniting with the fæculent matter of the chyme when the chylous precipitation has taken place, gradually loses its living, and acquires its chemical properties.

These changes in its nature have taken place when chylification has been accomplished, and when nothing remains of the chyme but its fæculent parts; it is then that the bile acts by its resin and by its alkali, by its bitterness and yellowness; and where the excrementitious canal, consisting of the cæcum, colon, and rectum, is its proper seat; the increased magnitude of this part is not only admirably fitted for the putrid fermentation which the fæculent matter there undergoes, but the smoothness of its surface (totally unlike the chymous canal either in fabric or design) is favourable to the expulsion of its putrid contents out of the system, as deleterious and foreign; whenever cystic bile is employed, it is rather to stimulate than to separate, to produce expulsion rather than assist chylification. Cystic bile therefore does in the chymous canal what hepatic bile does in the excrementitious.

It is to these fluids that Dr. Rush has ascribed the faculty of assisting chylification, by mixing with the chyle in order that it may be absorbed, to give tone to the intestines, &c.

Equally erroneous are his conclusions respecting the office of the *pancreas,* or the affinity which he supposes it bears in point of use to the salivary glands; the especial use of the salivary glands, after the food has been broken down with respect to mass by the action of the teeth, is destined to assist them in comminuting those parts into smaller particles, and otherwise to eliminate the sensible and chemical qualities which food contains, and with which it became involved; the saliva bereaves acids of their acidity, alkalis of their acrimony, and to a certain and limited limited extent it blunts the asperity of the ingesta in general rendering them bland and mild.

With respect to the pancreatic juice the case is precisely reversed, instead of being received with the chyle into the system, as the saliva is with the ingesta received into the stomach, forming a portion of the chyme, the pancreatic juice after it has performed the office of precipitating the chyle from the fæces, unites with them, acting probably by virtue of the chemical parts of which it is composed, and which by analysis have been ascertained, assisting the decomposed bile, and feculent matter of the chyme, in stimulating the feculent canal, from whence the whole is expelled as deleterious and foreign.

Having briefly answered the particular points on which Dr. Rush has principally dwelt, for the establishment of his new ideas on the hepatic system, it cannot be reasonably expected that I should expatiate on the false facts which he has advanced, or the false conclusions which he has drawn from them. I have neither time nor opportunity at present for such a task; I shall therefore briefly notice the use for which the spleen is probably designed.

I was naturally led to this enquiry, from the phænomena which I have described in the process of chylification. If this process was perpetually taking place, and the organs subservient to it were in perpetual action, it is very probable that the Almighty, in the formation of animals in general, would have supplied them with powers adequate to that end: it is far otherwise; the final cause of the higher order of animals in general is not the gratification of the appetite alone; hence arises the occasional instead of the constant calls of hunger; it is to satisfy these calls that animals are led to take food, in order to supply the wants, and prevent the waste of the system. It is from this occasional want that they are led to take a larger quantity of food at particular times; and such is the indigent condition to which all are doomed, that the quality is often neither congenial to the nature of their frames in general, nor adapted to the ordinary power of the chylopoetic organs to assimilate in particular; most animals therefore are supplied with a provision by means of which this imperfection may be obviated; by which an extraordinary quantity of blood may be furnished, at those times when it is more immediately wanted for the purpose of supplying the stomach and pancreas with gastric and pancreatic juice, and finally the liver with bile.

If the construction of the spleen be examined, the probability that it subserves such an office will be apparent; it it possesses no excretory duct, nor secretes any fluid, the blood which flows into it consequently undergoes no alteration, so that the blood in the splenic vein is nearly the same as the blood contained in the splenic artery; and, finally, that it is not essentially necessary to the preservation of the animal œconomy is proved, because it has been often removed by art, and occasionally absent by nature, without producing any effects apparently deleterious. I am therefore led to conclude also, that it is only an organ like the pancreas and liver, of an auxiliary nature.

It is from this negative state of subsistence, and of subordination, of knowing what it is not, that we are in some degree led to find out what it is; that we are led to suppose it is destined to afford assistance to the chylopoetic organs in general, by supplying them with a larger quantity of blood, for the production of gastric or pancreatic juice and bile, at those times that they are particularly wanted.

It is difficult to say how this effect is produced; from seeing however the sympathy which prevails between different parts of the system, whose actions are designed to the same end, I am disposed to believe that it is produced by a sympathetic and not a mechanical cause. I feel myself greatly indebted to Dr. Haighton for the experiments which he has so ably made upon this organ, and which have thrown considerable light on the subject; they go to prove, that whenever the stomach is full, the pressure which it produces on the spleen, not only prevents the passage of blood from the splenic artery through that organ, but that it does actually cause an increased accumulation in the vessels, which the splenic trunk gives out to the stomach and pancreas. If this be the case the liver must also receive a larger quantity of blood from the vena portæ for the secretion of bile, as it is principally made up of the veins which return the blood from those organs.

Whether the spleen is to be considered as an auxiliary organ to the chylopoetic viscera in particular, or, according to Dr. Rush, as a reservoir to the vascular system in general, I shall not pretend to decide. The opinion is a plausible and an ingenious one; there are many objections to it which I have not time to enumerate; I must therefore conclude with an apology for the hasty manner in which I write, and for the brief analysis which I now give of what I have stated more at large in another work.

I am, &c.

RICHARD SAUMAREZ.

*Kennington, Surry,*

